# Age-Dependent Regulation of Acetylcholine Release at the Neuromuscular Junction Mediated by GABA

**DOI:** 10.3390/cells14241949

**Published:** 2025-12-09

**Authors:** Egor Nevsky, Guzel Sibgatullina, Dmitry Samigullin, Artem Malomouzh, Vladimir Parpura, Konstantin Petrov

**Affiliations:** 1 Kazan Institute of Biochemistry and Biophysics, FRC Kazan Scientific Center of RAS, 420111 Kazan, Russia; nevskywissen@gmail.com (E.N.); artur57@list.ru (A.M.); 2 Department of Human and Animal Physiology, Institute of Fundamental Medicine and Biology, Kazan Federal University, 420008 Kazan, Russia; 3 Department of Radiophotonics and Microwave Technologies, Kazan National Research Technical University Named After A.N. Tupolev-KAI, 420111 Kazan, Russia; 4 International Translational Neuroscience Research Institute, Zhejiang Chinese Medical University, Hangzhou 310053, China; 5 Arbuzov Institute of Organic and Physical Chemistry, FRC Kazan Scientific Center of RAS, 420111 Kazan, Russia

**Keywords:** neuromuscular junction, acetylcholine release, γ-aminobutyric acid, GABA_B_ receptor, newborn mice, ontogenesis

## Abstract

γ-Aminobutyric acid (GABA) is the main inhibitory neurotransmitter in the central nervous system. However, GABA receptors, notably at the neuromuscular junction (NMJ), have also been identified in the peripheral nervous system. Here, we studied GABA_B_ receptor (GABA_B_–R)-mediated regulation of acetylcholine (ACh) release in mouse NMJs during early postnatal development. The results revealed that, depending on the age of the mice, the activation of GABA_B_–R had the opposite effect on ACh release. At the NMJ in mice on the second postnatal (P2) day, the GABA_B_–R blocker CGP 55845 (5 μM) significantly increased the level of ACh release, whereas the GABA_B_–R agonist baclofen (10 μM) decreased ACh release. In P14-aged mice, CGP 55845 decreased ACh release, while the application of baclofen significantly increased the release. At the NMJ of P14 mice, the mechanism of the ACh release-potentiating effect of GABA_B_–R activation involves N-type calcium ion channels and small-conductance calcium ion-activated potassium ion channels.

## 1. Introduction

γ-Aminobutyric acid (GABA) is a key inhibitory neurotransmitter in the central nervous system. GABA acts via the activation of two types of receptors, GABA_A_ (ionotropic) and GABA_B_ (metabotropic). The activation of GABA_A_ receptors, after the postnatal shift [1], results in an increase in an inward chloride ion current that induces hyperpolarization of the neuron and blocks action potential generation [2]. Metabotropic GABA_B_ receptors (GABA_B_–Rs) are G protein–coupled receptors that trigger intracellular signal transduction mainly via interactions with G_i/o_ proteins [3]. The activation of GABA_B_–R can modulate the release of neurotransmitters through the inhibition of voltage-gated Ca^2+^ channels, inward-rectifying potassium channels (GIRKs) and two-pore-domain TREK-2 potassium channels [4,5].

There is some evidence that GABA_B_–Rs are involved in the regulation of neurotransmitter release not only in the central nervous system but also at neuromuscular junctions (NMJs) [6]. At vertebrate NMJs, the main neurotransmitter is acetylcholine (ACh), which is released from motor nerve terminals and activates postsynaptic nicotinic acetylcholine receptors (nAChRs) of the muscle type. The level of ACh released by motor neurons is not constant and can be dynamically regulated via different pathways [7,8,9,10,11,12,13,14,15,16].

The activation of the ACh release regulatory pathway, which includes GABA, has been previously described in the NMJs of adult mice with inhibited cholinesterase (ChE). After ChE inhibition, the activation of the α7 nAChRs, expressed on plasmalemmas of terminal Schwann cells (TSCs), by ACh spillover induced the secretion of GABA. GABA acts on GABA_B_–Rs on presynaptic terminals to reduce ACh release [17,18]. In adult mice with active ChE, activation of this α7/GABA_B_–R regulatory pathway was not detected. Recently, the application of the GABA_B_–R antagonist CGP 55845 was shown to increase the probability of ACh release in the diaphragm muscle of mice on the second postnatal (P2) day [18]. This effect of GABA_B_–R blockade in newborn mice was associated with intact ChE activity. Thus, endogenous GABA is released at newborn NMJs and affects cholinergic neurotransmission without ChE inhibition. However, the involvement of α7 nAChRs in the GABA_B_–R-mediated pathway in neonates has not been studied [18].

In the present study, we further investigated GABA_B_–R-mediated regulation of ACh release in mouse NMJs during early postnatal development. At P2, the α7/GABA_B_–R regulatory pathway decreases ACh release in the mouse *levator auris longus* (LAL) muscle. However, we also found that at P14, GABA increases ACh release in the NMJs of the LAL and that α7 nAChRs are not involved. Thus, in the first weeks of postnatal development, the effect of GABA on ACh release is reversed from depression to augmentation. Both effects of GABA on ACh release are mediated by the activation of GABA_B_–R.

## 2. Materials and Methods

### 2.1. Ethical Approval and Animals

Animal care and experiments were carried out in accordance with the NIH Guide for the Care and Use of Laboratory Animals and the EU Directive 2010/63/EU. The protocol for the experiments was approved by the Animal Care and Use Committee of the FRC Kazan Scientific Center of the Russian Academy of Sciences (protocol #23/7; 12 May 2023). All efforts were made to minimize animal suffering. BALB/c mice were kept in sawdust-lined plastic cages in a well-ventilated room at 20–22 °C under a 12 h light/dark cycle at 60–70% relative humidity and given ad libitum access to food and water.

The studies were performed on neuromuscular preparations of LAL [19] isolated from the animals at two (P2) and fourteen (P14) days after birth and from the animals at two months of age (P60). The animals were deeply anesthetized with isoflurane and then quickly decapitated. Animals of each age were not intentionally randomized, and the investigators were not blinded to the nature of the chemicals used.

### 2.2. Chemicals

The GABA_B_–R blocker CGP 55845 (Cat. #SML0594), the α7 nAChR blocker methyllycaconitine (MLA; Cat. #M168), and the phospholipase C inhibitor U-73122 (Cat. #U6756) were purchased from Sigma—Aldrich, St. Louis, MO, USA. The SK-type calcium-activated potassium channel blocker apamin (Cat. #1652), the inhibitor of adenylyl cyclase SQ 22536 (Cat. #1435/10), and the inhibitor of the N-type calcium channel ω-conotoxin GVIA (Cat. #1085/250U) were purchased from Tocris Cookson Inc., Bristol, UK. The GABA_B_–R agonist baclofen (Cat. #B3343) was purchased from Tokyo Chemical Industry, Tokyo, Japan. 3-(N-butylethanimidoyl)-4-hydroxy-2H-chromen-2-one was purchased from Syntrade-Kazan, Kazan, Russia. The recycling dye N-(3-triethylammoniumpropyl)-4- (4- (dibutylamino) styryl) pyridinium dibromide (FM1-43, Cat. #T-35356) was purchased from Thermo Fisher Scientific, Waltham, MA, USA.

The concentrations of agonists and antagonists reported in this study were obtained from the literature data that have shown a physiological response due to selective pharmacological action: CGP 55845 (5 μM) [20], baclofen (10 μM) [21], MLA (10 μM) [22], U-73122 (5 μM) [23], apamin (100 nM) [24], SQ 22536 (100 μM) [25] and ω-conotoxin GVIA (1 μM) [26].

### 2.3. Electrophysiological Recordings

Nerve–muscle preparations of LAL were pinned in translucent chambers and perfused at a rate of 2–3 mL min^−1^ with oxygenated (95% O_2_, 5% CO_2_) Krebs–Ringer solution containing 154 mM NaCl, 5 mM KCl, 2 mM CaCl_2_, 1 mM MgCl_2_, 5.0 mM HEPES buffer and 11 mM glucose (pH 7.4). End-plate potentials (EPPs) and miniature EPPs (mEPPs) were recorded at 20–22 °C via a standard glass microelectrode (resistance 10–15 MΩ) filled with 3 M KCl. Since µ-conotoxin GIIIB is not effective in newborns [27], to prevent the generation of action potentials and muscle contractions during EPP/mEPP recording, transverse cutting of muscle fibers was used [28]. The motor nerve was stimulated via a suction electrode with supramaximal current pulses (duration 0.1 ms) with a frequency of 0.5 Hz. Recordings were made in 5–10 end plates before treatment and in 5–10 other end plates after treatment. At each end plate, 50 EPPs and 50 mEPPs were recorded. The quantal content (QC) of the EPPs was calculated by dividing the mean EPP amplitude by the mean mEPP amplitude recorded in the same end plate. Only fibers with a stable resting membrane potential at −40 ± 2 mV were analyzed. To correct the QC value for the nonlinearity of the EPP/mEPP amplitude summation, Martin’s correction was used [29].

Synaptic responses were recorded via an Axoclamp 900A amplifier, digitized via Digidata 1440A (Molecular Devices, San Jose, CA, USA) and analyzed via pClamp v 10.4 software (Molecular Devices, San Jose, CA, USA).

### 2.4. Fluorescence Assays of Acetylcholine Release

Optical monitoring of changes in the process of synaptic vesicle exocytosis was carried out using the fluorescent dye FM1-43, which interacts with the external leaflet of the plasma membrane and is then taken up into synaptic vesicles by endocytosis. Motor nerve stimulation causes dye release from dye-preloaded synaptic vesicles [30].

FM1-43 was loaded into synaptic vesicles at 30 Hz (for 3 min), thereby inducing massive exocytosis-endocytosis. FM1-43 (7 μM) was present in the incubation solution 3 min before stimulation, during stimulation and 5 min after the end of stimulation. The preparations were then washed for 40 min with Ringer-Krebs solution. FM1-43-loaded nerve terminals were then stimulated at 30 Hz to induce massive synaptic exocytosis, resulting in the release of dye from vesicles (FM1-43 destaining). Muscle contractions were blocked by the skeletal muscle myosin inhibitor 50 μM 3-(N-butylethanimidoyl)-4-hydroxy-2H-chromen-2-one [31].

The fluorescence intensity of FM1-43 dye in nerve terminals was calculated via the following formula: (ΔF = F_ROI_ − F_back_), where F_ROI_ is the average pixel intensity at a particular synapse and F_back_ is the fluorescence intensity of the background component defined in a 4 × 30 μm^2^ region outside the nerve terminals. The initial value of nerve terminal fluorescence, ΔF_max_, was taken as 100%, and all other values were normalized to it as described previously [32].

The fluorescence signal was captured via an imaging system that included an Olympus BX-51 microscope equipped with a ×40 water-immersion objective (Olympus, Tokyo, Japan). The unloading of FM1-43-labeled synapses was recorded with a high-sensitivity RedShirtImaging NeuroCCD-smq camera (RedShirtImaging, Decatur, GA, USA). A Polychrome V light source (Till Photonics, Munich, Germany) was used at a wavelength of 488 nm. For data acquisition, we utilized Turbo-SM software (version 4.2.0.2, RedShirtImaging, Decatur, GA, USA).

### 2.5. Statistics

OriginLab 2021b software was used for statistical analysis. The data are presented as the means ± standard errors of the means (SEMs). The sample size (n) is the number of independent neuromuscular junctions obtained from 4 to 6 individual animals; ‘n’ is indicated in each figure legend.

The sample size required for an individual set of experiments was preassessed via power analysis (set at 80% and α = 0.05). There were no exclusions from outliers. Significance was assessed by the Mann–Whitney U test. * *p* < 0.05, ** *p* < 0.01 and *** *p* < 0.001 were considered statistically significant compared with control values, and # *p* < 0.05, ## *p* < 0.01 and ### *p* < 0.001 were considered statistically significant compared with prior treatment with inhibitor/blocker values.

## 3. Results

### 3.1. GABA_B_–Rs Mediate the Modulation of EPP Quantal Content in Newborn, Young and Adult Mice

**Figure 1:**
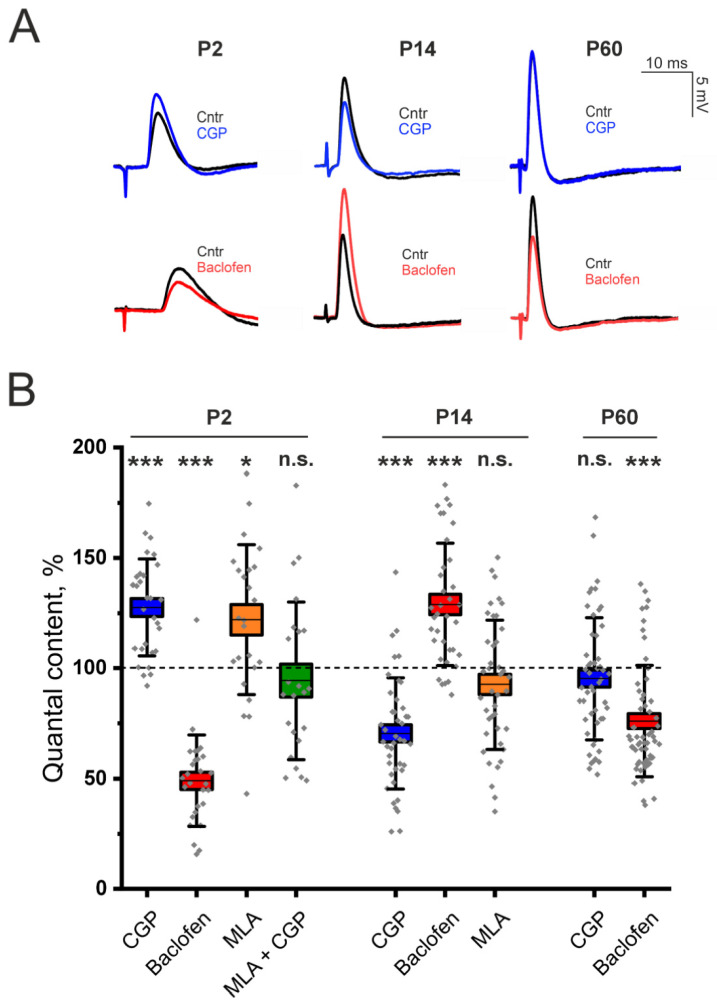
Involvement of GABA_B_–R in the regulation of ACh at LAL NMJs in neonatal (P2), young (P14) and adult (P60) mice. (**A**)—Representative EPPs recorded at the NMJs in the control (Cntr) and after the application of the GABA_B_–R blocker CGP 55845 (CGP, 5 μM) or the GABA_B_–R agonist baclofen (10 μM) at different stages of ontogenesis. (**B**)—Effects of GABA_B_–R ligands (CGP 55845 or baclofen) and an α7 nAChR antagonist (MLA, 10 nM) on the QC of EPPs upon 0.5 Hz motor nerve stimulation. The value of the QC in the control was taken as 100%. The data are presented as box plots: gray diamonds, relative changes in individual experiments; horizontal lines, M; whiskers, SD; boxes, SEM for the entire series of experiments (n = 28–56). n.s.—not significant, * *p* < 0.05 and *** *p* < 0.001—Mann–Whitney U test between the control and individual chemical groups.

We found that, e the application of the GABA_B_–R blocker CGP 55845 (5 μM) caused an increase in EPP QC of 33 ± 7% (*p* < 0.001) in the LAL of P2-aged mice, whereas the GABA_B_–R agonist baclofen (10 μM) caused a decrease in the QC of EPP of 50.9 ± 3.9% (*p* < 0.001; Figure 1). Hence, at the NMJs of P2-aged mice, stimulation of the motor nerve, in addition to ACh release, causes the secretion of GABA and the activation of GABA_B_–R, which triggers depression of ACh release.

Next, we studied the involvement of the α7 nAChR in the GABA_B_–R-mediated pathway of ACh release regulation in neonates. In the LAL muscle, the α7 nAChR blocker MLA (10 nM) had an effect similar to that of CGP 55845 (now on interchangeably used with the abbreviation CGP). Blockade of α7 nAChR significantly increased the QC of EPPs by 21.9 ± 6.9% (*p* < 0.05) in P2-aged mice (Figure 1). However, after the blockade of α7 nAChR, the blockade of GABA_B_–R with CGP 55845 had no effect on the QC of EPP (Figure 1). Thus, the pathway of ACh release regulation, mediated by α7/GABA_B_–R, is active in newborn animals at P2.

Unlike in neonates, in the LAL muscles of adult two-month-old (P60) mice, blockade of GABA_B_–R with CGP (5 μM) had no effect on the QC of the EPP. However, the application of the GABA_B_–R agonist baclofen (10 μM) significantly decreased the QC of EPP by 23.9 ± 3.4% (*p* < 0.001; Figure 1). The absence of the effect of CGP along with the decrease in the QC of the EPP upon application of baclofen were previously shown for the rat diaphragm muscle [6]. Thus, in adults, the GABA_B_–R-mediated pathway is less active than it is in neonates.

It can be assumed that the “silencing” of the pathway of ACh release regulation mediated by α7/GABA_B_–R already occurs in the neonatal period. Most likely, at the end of the process of remodeling, e.g., the transition of polyneuronal to mononeuronal innervation of the muscle fiber. Postnatal maturation of the NMJ occurs in several stages. However, it is generally accepted that by postnatal day 14 (P14), the morphology of the NMJ is mature [33].

Indeed, our experiments revealed that at P14, NMJs of LAL show no signs of downregulation of ACh release through the GABA_B_–R mediated pathway. We found the opposite effect of GABA_B_–R activation on ACh release. The application of the GABA_B_–R antagonist CGP 55845 (5 μM) decreased the EPP QC by 29.5 ± 3.9% (*p* < 0.001) in the LAL of P14 mice (Figure 1). The application of the GABA_B_–R agonist baclofen (10 μM) significantly increased the QC of EPP by 29.4 ± 6.3% (*p* < 0.001). Importantly, the blockade of α7 nAChR with MLA (10 nM) had no effect on EPP QC in P14 NMJs (Figure 1). Thus, α7 nAChR does not participate in the GABA_B_–R-mediated pathway of increasing ACh release.

### 3.2. GABA_B_-R-Mediated Potentiation of ACh Release at the NMJ of Young Mice Occurs via the Inhibition of N-Type Calcium Channels and Small-Conductance Calcium Ion-Activated Potassium Ion Channels

**Figure 2:**
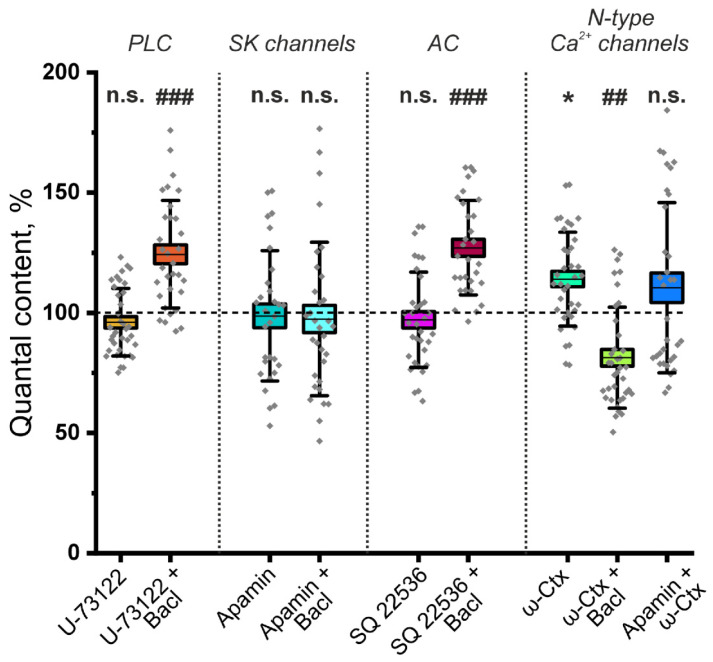
The mechanism of the ACh release-potentiating effect of GABA_B_–R activation at NMJs of young (P14) mice. Effect of the GABA_B_–R agonist baclofen (Bacl, 10 μM) on the QC of EPPs against the background of the phospholipase C (PLC) inhibitor U-73122 (5 μM), the small-conductance calcium ion-activated potassium ion (SK) channel blocker apamin (100 nM), the adenylate cyclase (*AC*) inhibitor SQ 22536 (100 μM) and the N-type Ca^2+^ channel blocker ω-conotoxin GVIA (ω-Ctx, 1 μM). The values of the QC in the control were taken as 100%. In the experiments with Bacl, the values obtained in the presence of blockers were taken as 100%. In the Apamin + ω-Ctx experiments, the values obtained in the presence of blocker SK channels were taken as 100%. The data are presented as box plots: gray diamonds, relative changes in individual experiments; horizontal lines, M; whiskers, SD; boxes, SEM for the entire series of experiments (n = 38–43). n.s.—not significant and * *p* < 0.05 vs. control values; ## *p* < 0.01 and ### *p* < 0.001 vs. values for Bacl compared with the effect of preincubation with blockers or inhibitors (Mann–Whitney U test).

What could be the mechanism by which increasing ACh release is mediated by the activation of GABA_B_–R? In adult rats, GABA application reduces the QC of EPPs via the participation of phospholipase C (PLC) [6]. However, the activation of PLC can also lead to an increase in ACh release. It has been shown that the activation of PLC can decrease tetanic depression at the frog NMJ [34]. However, we have shown that application of the PLC inhibitor U-73122 (5 μM) did not lead to a significant decrease in the effect of baclofen (10 μM) on ACh release (Figure 2). After the inhibition of PLC, baclofen increased the EPP QC at the NMJ of P14 mice by 31.3 ± 5.4% (*p* < 0.001). Thus, PLC is not involved in the pathway of increased ACh release mediated by the activation of GABA_B_–R.

The activation of GABA_B_–R can increase neurotransmitter release through a decrease in adenylyl cyclase (AC) activity, which reduces the activity of small-conductance calcium ion-activated potassium ion (SK) channels [35]. Indeed, the blockade of SK channels by apamin (100 nM) did not significantly affect ACh release; however, it prevented the increase in the EPP QC caused by baclofen (Figure 2). Decreased potassium outflow through SK channels increases the duration of nerve action potentials, leading to increased neurotransmitter secretion [36]. To test the involvement of AC, its selective inhibitor SQ22536 (100 μM) was used. Pretreatment with SQ22536 did not prevent the increase in QC induced by baclofen, as the QC increased by 27.2 ± 4.6% (*p* < 0.001). Thus, SK channels but not ACs are involved in the pathway of increasing the EPP QC, which is mediated by the activation of GABA_B_–R.

The activation of G protein-coupled receptors causes the dissociation of Gα and Gβγ heterodimers [37]. Gβγ can directly bind to the Cav2 subunit of N-, P/Q- and R-type voltage-gated calcium ion channels. The interaction of Cav2 with Gβγ caused channel inhibition [38]. It can be speculated that the GABA_B_–R activation may decrease the activity of SK potassium channels if they are coupled to Cav2-containing calcium ion channels. A similar mechanism of GABA-mediated excitation of release involving GABA_B_–R, N-type calcium channels coupled to conductance potassium ion (BK) channels has been previously described in rat retinal ganglion cells [39].

To test the hypothesis of the involvement of N-type calcium channels, we studied the effect of baclofen on the QC of EPP after the blockade of these calcium channels with the ω-conotoxin GVIA. The application of ω-conotoxin GVIA (1 μM) alone significantly increased the EPP QC to 28.9 ± 9.3% (*p* < 0.01) in P14 mice. However, the application of baclofen after the blockade of N-type calcium ion channels did not increase but rather significantly decreased the QC at the NMJs of P14 mice (Figure 2). Importantly, the effect of ω-conotoxin GVIA on the QC of EPP was not observed after the blockade of SK channels with apamin (Figure 2). Thus, at P14, the activation of GABA_B_–R leads to the inhibition of N-type calcium ion channels, which is likely functionally coupled with SK channels. A decrease in potassium outflow prolongs the process of depolarization of nerve endings, which leads to an increase in the release of ACh. Notably, if N-type calcium channels are blocked with the ω-conotoxin GVIA, the “silencing” pathway of ACh release downregulation can be activated at the NMJs of P14-aged mice. Thus, it can be postulated that there is a mechanism for the occlusion of this pathway when the GABA_B_–R pathway of ACh release augmentation is active.

### 3.3. Fluorescence Assays of Acetylcholine Release/Regulated Exocytosis from Motor Nerves of Newborn (P2) or Young (P14) Mice After GABA_B_–R Inhibition

To confirm the bidirectional effects of GABA_B_–R blockade on ACh release at P2 and P14, we performed experiments to assess the extent regulated exocytosis and hence ACh release via the fluorescent marker of recycled synaptic vesicles FM 1-43.

**Figure 3:**
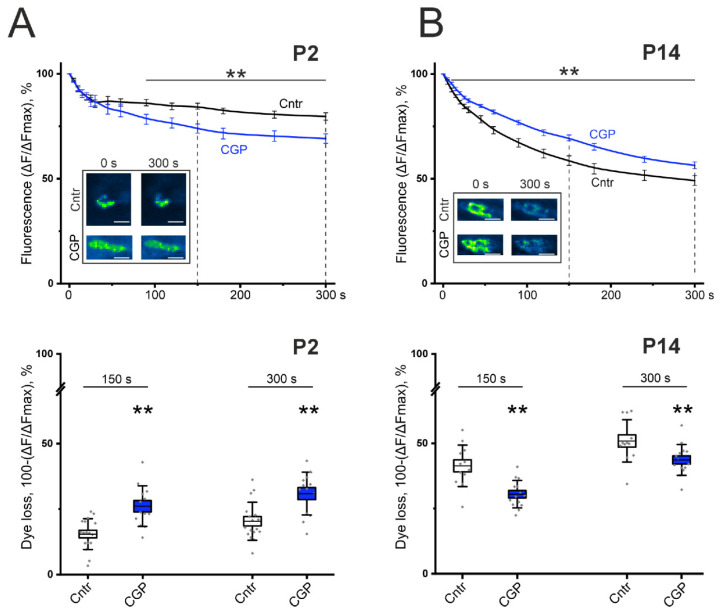
Effect of the GABA_B_-R blocker CGP 55845 (5 μM) on exocytotic dye release from FM1-43-preloaded motor nerves of the LAL of newborn (P2, panel (**A**)) and young (P14, panel (**B**)) mice. (**Top**)—kinetics of exocytotic FM 1-43 dye release from motor nerve terminals in control (Cntr, black) and CGP-treated nerve-muscle preparations (CGP, blue). The inset shows typical fluorescence images of NMJs before the onset (0) and at 300 s of stimulation in the control and in the presence of CGP 55845. Scale bars, 15 µm. (**Bottom**)—the portion of dye fluorescence loss during 150 s and 300 s of stimulation at 30 Hz in control (Cntr; n = 12–17) and CGP-treated nerve terminals (n = 13–14). ** *p* < 0.01 vs. control values.

In control experiments on the LAL of P2 mice, stimulation of the motor nerve led to a decrease in the fluorescence level to 79.7% of the initial level at 5 min (Figure 3). In the presence of a GABA_B_-R blocker (CGP 55845, 5 μM), the rate of FM 1-43 destaining of the nerve terminal accelerated (significantly from 90 s), thereby releasing 9.1 ± 2.5% (*p* < 0.01) more dye at 150 s than in the control and 8.5 ± 4.1% (*p* < 0.01) more dye at 300 s; after 5 min, the fluorescence level decreased to 69.1% (*p* < 0.01) (Figure 3). These data indicate an increase in the process of evoked/regulated exocytosis of synaptic vesicles after blockade of GABA_B_–R.

At the age of P14, the control value of FMs 1-43 destaining of the LAL nerve terminals by the end of 5 min was 49.1% (Figure 3). The application of CGP 55845 (5 μM) did not increase, but rather decreased the rate of destaining and released 10.2 ± 3.4% (*p* < 0.01) less dye at 150 s and 6.7 ± 3.5% (*p* < 0.01) less dye at 300 s than the control. A statistically significant difference in the dynamics of destaining was reliably detected from the 10th second of stimulation; after 5 min, the fluorescence level decreased to 56.4% (*p* < 0.01; Figure 3). These data may indicate a weakening of the process of evoked exocytosis of synaptic vesicles during blockade of GABA_B_–R.

Thus, the fluorescence microscopy data confirmed the data of electrophysiological experiments on the bidirectional effect of GABA_B_–R activation on ACh release at P2 and P14.

## 4. Discussion

GABA is an inhibitory neurotransmitter in the central and peripheral nervous systems. Therefore, pathways regulating neurotransmitter secretion that involve GABA receptors are aimed mainly at decreasing release. However, a number of studies have shown that the activation of GABA_B_–R can increase secretion through the inhibition of BK-type [39], SK-type [35], or both types of these potassium ion channels [40].

**Figure 4:**
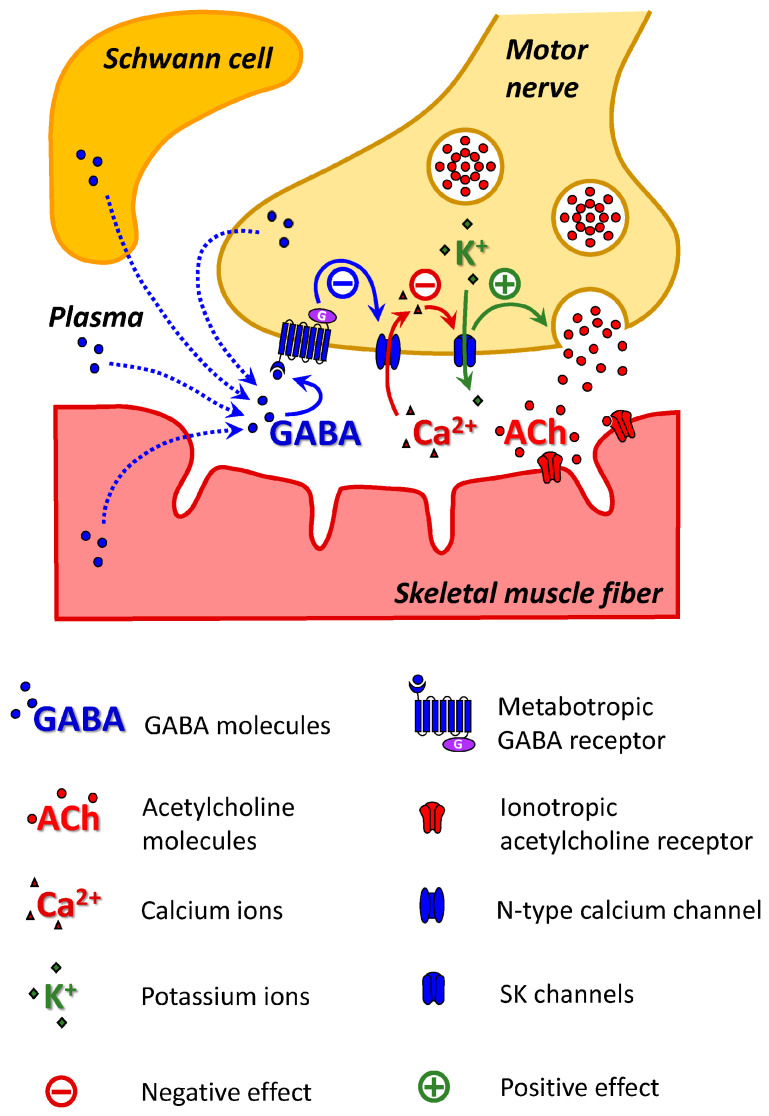
Scheme of the GABA_B_–R-mediated ACh release augmentation pathway at NMJs in P14 mice. SK channels on the membrane at the motor nerve ending are activated by Ca^2+^ entering through N-type calcium channels. The activation of GABA_B_–R reduces the activity of N-type calcium channels and, consequently, the activity of SK channels. A decrease in potassium outflow prolongs the process of depolarization of the nerve ending, which leads to an increase in the release of ACh.

The initial goal of this study was to test the hypothesis that the α7/GABA_B_–R pathway downregulates ACh release at the NMJ during early ontogenesis. The effect of the α7/GABA_B_–R-mediated decrease in ACh release was detected at the NMJs of the mice on the second day after birth. However, unexpectedly, during postnatal development, another GABA_B_–R-mediated pathway that increases ACh release and does not involve the α7 nAChR is also active. On the 14th day after birth, activation of GABA_B_–R has been shown to decrease the activity of N-type voltage-gated calcium ion channels. The presence of this type of calcium ion channel in the neonatal NMJ has been previously described [41], but its role remains unknown. However, we have shown that N-type calcium ion channels are likely coupled with SK potassium ion channels. It is assumed that the inhibition of N-type calcium channels can decrease the activity of potassium ion channels, which can explain the increase in ACh release (Figure 4). The intracellular mechanisms of decreased ACh release at NMJs in P2 mice remain unknown and require further research. Thus, further studies employing genetic, molecular and cellular tools would help to unambiguously delineate the pathway underlying GABA_B_–R modulation of ACh release at the NMJ.

Muscarinic AChRs and adenosine receptors [42,43] are known to be involved in the regulation of ACh release at the newborn NMJ. In newborn animals, skeletal muscle fibers are innervated by several competing axons. During the first two weeks after birth, the NMJ remodeling process occurs, ending with the “victory” of one of the nerve endings and the elimination of others [44]. A high level of ACh release from a nerve terminal is one of the key factors determining the “winning” terminal [45]. The pathways of ACh release regulation activated by muscarinic AChRs or adenosine receptors provide competitive interactions between nerve endings. More active endings can directly “punish” less active endings by additionally suppressing ACh release or “rewarding” themselves [46].

The morphological effects of GABA_B_–R activation on early postnatal neuromuscular development have been previously described. The myelination of the motor nerve starts after the end of NMJ remodeling [47]. GABA_B_–Rs are expressed in premyelinating Schwann cells along the motor nerve and are downregulated from P0 to P15 [48]. Baclofen decreases the synthesis of myelin proteins in pure cultures of Schwann cells from neonatal rats [49]. Thus, activation of GABA_B_–R delays myelination of the motor nerve in the early postnatal period.

The origin of GABA is not fully understood. Sources can include nerve terminals, TSCs, muscle fibers, or blood plasma (Figure 4). There is immunohistochemical evidence of the presence of GABA, glutamate decarboxylase (the enzyme involved in the synthesis of GABA), GABA_B_–R, and GABA transporters (GAT-1, GAT-2) at NMJs in adult animals [18,50,51]. A comprehensive gene expression profile of TSCs and triceps brachii muscle in adult mice revealed that glutamate decarboxylases (Gad1 and Gad2) and GABA transporters (Best1, Slc6a1, Slc6a11 and Slc6a12) were enriched in TSCs [18]. Thus, GABA can be synthesized in the area of NMJs and released during neuromuscular synaptic transmission via GABA transporters and Bestrophin1, a Ca^2+^-activated anion channel [52].

The involvement of GABA in neuromuscular synaptic transmission in neonates has been described recently [18], and its physiological contribution is not fully understood. Petrov et al. reported that the GABA_B_–R blocker CGP 55845 significantly increases the decrease in the force of diaphragm muscle contraction in P1 mice ex vivo [18]. Thus, in the first days after birth, decreased ACh release can contribute to fatigue resistance.

**Figure 5:**
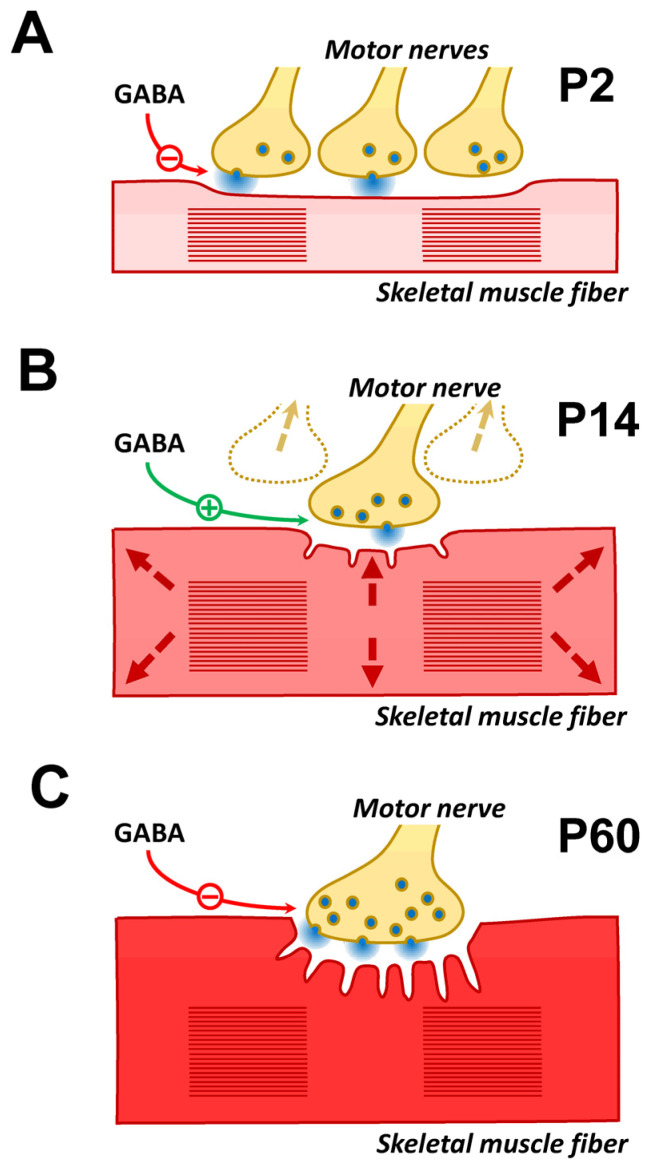
Hypotheses about the physiological role of GABA-mediated ACh release at different stages of the NMJ ontogeny. (**A**) P2-aged mice. At this age, each muscle fiber is innervated by several motor neurons that release ACh equally. Over the next few days, competition between nerve endings begins, with a gradual decrease in release from the “losing” endings and their complete elimination. It can be assumed that at the age when competition has not yet begun (P2), ACh release is excessive. Therefore, mechanisms exist that prevent unnecessary ACh consumption or “flooding” of the NMJ by excess ACh. (**B**) P14-aged mice. At this age, the process of NMJ remodeling is complete, and muscle mass growth begins (shown by arrows). To maintain the reliability of synaptic transmission, an increase in muscle fiber diameter is accompanied by an increase in the NMJ area. However, morphological changes in the NMJ take time. It can be hypothesized that increased ACh release is used as an additional, rapid mechanism to maintain the reliability of synaptic transmission. (**C**) P60-aged mice. At this age, the effect of endogenous GABA is not detected if ChE is active. However, the application of GABA or GABA_B_–R agonists causes a decrease in the QC of EPP. Under conditions of inhibited ChE, an effect of endogenous GABA similar to that of a GABA_B_–R agonist is detected. It can be assumed that this pathway for regulating ACh release remains active in adult animals, for example, for fine tuning of synaptic transmission by ACh spillover. The possibility that this GABA-mediated regulatory pathway is activated by natural ChE inhibitors cannot be ruled out. Natural ChE inhibitors are found both in some plants (physostigmine, caffeine, etc.) and certain snake venoms (fasciculin).

Mouse muscle nAChRs undergo a developmental switch in subunit composition in the first two weeks after birth [33]. Fetal muscle receptors are designed to function at low ACh concentrations. Therefore, they have high sensitivity to ACh but are easily desensitized in the presence of high concentrations [53]. This contrasts with adult receptors, which are ideally tailored for robust and reproducible high-frequency neuromuscular transmission at mature synapses, where ACh reaches millimolar concentrations [54]. We hypothesize that a partial reduction in release via this GABA-mediated pathway can prevent ACh from overrun in the synaptic cleft during polyneuronal innervation (Figure 5A). In the first days after birth, before the competition between nerve endings begins, full rerelease of ACh from 3–4 nerve endings may be too excessive. A partial reduction in release via this GABA-mediated pathway can prevent excess ACh and desensitization. Moreover, for example, excessive recruitment of a readily releasable pool of synaptic vesicles can be prevented. This hypothesis is consistent with results previously obtained in adult mice [18]. As mentioned above, in adult mice, the effect of endogenous GABA was detected only when ChE was inhibited (Figure 5C). An excess of ACh in the synaptic cleft due to ChE inhibition triggered a decrease in ACh release, which was mediated by GABA_B_–R. Notably, GABA_B_–R blockade reduces fatigue in diaphragm muscles treated with a ChE inhibitor [18]. Thus, the effect of the GABA_B_–R blocker on muscle fatigue is similar in neonatal mice with intact ChE and in adults with inhibited ChE. However, the participation of this pathway in the process of communication between competing nerve endings cannot be excluded.

In the first two postnatal weeks, the NMJ of newborn animals undergoes cardinal structural and functional reorganizations. By the age of P14, the remodeling process is complete, and the morphology of all NMJ components generally corresponds to that of adult animals, but there are also differences [33,55]. During this period of postnatal development, the diameter and length of muscle fibers rapidly increase, which is accompanied by a change in their electrical properties. Intercalary growth of synaptic contacts is required to maintain the reliability of synaptic transmission. This process is crucial for NMJ enlargement during development and in response to increased neuromuscular activity [56]. This ensures that the pre-synaptic and post-synaptic specializations grow and maintain a precise correspondence as the muscle fiber itself grows. During this period, new postsynaptic folds and new sites of ACh release are intensively formed in NMJ synapses [57]. The activation of the GABA-mediated pathway of ACh release augmentation correlates with this “adolescent” period of postnatal development. It can be assumed that this pathway contributes to ensuring the safety of synaptic transmission during the period of intercalary growth of NMJs (Figure 5B).

## 5. Conclusions

GABA may be an important, previously unaccounted player in the process of postnatal maturation of the NMJ. In this study, we describe the bidirectional regulation of ACh release at the NMJ in newborn and young animals, which is mediated by metabotropic GABA receptors. During postnatal development, some GABA-mediated ACh release regulatory pathways are activated, whereas others are occluded. This may indicate that each process plays a significant physiological role.

## Data Availability

All data and materials are available in the article or upon request to the corresponding authors.

## Abbreviations

The following abbreviations are used in this manuscript:

ACh	Acetylcholine
AC	Adenylyl cyclase
BK	Big conductance potassium ion channels
ChE	Cholinesterase
Cntr	Control
EPP	End-plate potential
FM1-43	N- (3-triethylammoniumpropyl)-4- (4- (dibutylamino) styryl) pyridinium dibromide
GABA	γ -aminobutyric acid
GABA_B_–R	GABAB receptor
LAL	Levator auris longus
NMJ	Neuromuscular junction
MLA	Methyllycaconitine
nAChR	Nicotinic acetylcholine receptor
PLC	Phospholipase C
P	Postnatal day
QC	Quantal content
SK	Small-conductance calcium ion-activated potassium ion channels
mEPP	Miniature end-plate potential
SD	Standard deviation
SE	Standard error
TSC	Terminal Schwann cell

## References

[1] Peerboom C., Wierenga C.J. (2021). The postnatal GABA shift: A developmental perspective. Neurosci. Biobehav. Rev..

[2] Siucinska E. (2019). Γ-Aminobutyric acid in adult brain: An update. Behav. Brain Res..

[3] Shaye H., Stauch B., Gati C., Cherezov V. (2021). Molecular mechanisms of metabotropic GABAB receptor function. Sci. Adv..

[4] Gassmann M., Bettler B. (2012). Regulation of neuronal GABA(B) receptor functions by subunit composition. Nat. Rev. Neurosci..

[5] Deng P.Y., Xiao Z., Yang C., Rojanathammanee L., Grisanti L., Watt J., Geiger J.D., Liu R., Porter J.E., Lei S. (2009). GABA(B) receptor activation inhibits neuronal excitability and spatial learning in the entorhinal cortex by activating TREK-2 K+ channels. Neuron.

[6] Malomouzh A.I., Petrov K.A., Nurullin L.F., Nikolsky E.E. (2015). Metabotropic GABAB receptors mediate GABA inhibition of acetylcholine release in the rat neuromuscular junction. J. Neurochem..

[7] Tomàs J., Santafé M.M., Garcia N., Lanuza M.A., Tomàs M., Besalduch N., Obis T., Priego M., Hurtado E. (2014). Presynaptic membrane receptors in acetylcholine release modulation in the neuromuscular synapse. J. Neurosci. Res..

[8] Petrov K.A., Nikolsky E.E., Masson P. (2018). Autoregulation of Acetylcholine Release and Micro-Pharmacodynamic Mechanisms at Neuromuscular Junction: Selective Acetylcholinesterase Inhibitors for Therapy of Myasthenic Syndromes. Front. Pharmacol..

[9] Santafe M.M., Priego M., Obis T., Garcia N., Tomàs M., Lanuz M.A., Tomàs J. (2015). Adenosine receptors and muscarinic receptors cooperate in acetylcholine release modulation in the neuromuscular synapse. Eur. J. Neurosci..

[10] Ribeiro J.A., Cunha R.A., Correia-de-Sá P., Sebastião A.M. (1996). Purinergic regulation of acetylcholine release. Prog. Brain Res..

[11] Sousa-Soares C., Noronha-Matos J.B., Correia-de-Sá P. (2023). Purinergic Tuning of the Tripartite Neuromuscular Synapse. Mol. Neurobiol..

[12] Giniatullin A.R., Mukhutdinova K.A., Petrov A.M. (2024). Mechanism of Purinergic Regulation of Neurotransmission in Mouse Neuromuscular Junction: The Role of Redox Signaling and Lipid Rafts. Neurochem. Res..

[13] Petrov A.M., Zakirjanova G.F., Kovyazina I.V., Tsentsevitsky A.N., Bukharaeva E.A. (2022). Adrenergic receptors control frequency-dependent switching of the exocytosis mode between “full-collapse” and “kiss-and-run” in murine motor nerve terminal. Life Sci..

[14] Gaydukov A., Bogacheva P., Tarasova E., Molchanova A., Miteva A., Pravdivceva E., Balezina O. (2019). Regulation of Acetylcholine Quantal Release by Coupled Thrombin/BDNF Signaling in Mouse Motor Synapses. Cells.

[15] Bogacheva P.O., Molchanova A.I., Pravdivceva E.S., Miteva A.S., Balezina O.P., Gaydukov A.E. (2022). ProBDNF and Brain-Derived Neurotrophic Factor Prodomain Differently Modulate Acetylcholine Release in Regenerating and Mature Mouse Motor Synapses. Front. Cell. Neurosci..

[16] Tarasova E., Bogacheva P., Chernyshev K., Balezina O. (2024). Quantal size increase induced by the endocannabinoid 2-arachidonoylglycerol requires activation of CGRP receptors in mouse motor synapses. Synapse.

[17] Petrov K.A., Girard E., Nikitashina A.D., Colasante C., Bernard V., Nurullin L., Leroy J., Samigullin D., Colak O., Nikolsky E. (2014). Schwann cells sense and control acetylcholine spillover at the neuromuscular junction by α7 nicotinic receptors and butyrylcholinesterase. J. Neurosci..

[18] Petrov K., Lenina O., Leroy J., Bernard V., Germain T., Truong C., Nurullin L., Sibgatullina G., Ohno K., Samigullin D. (2025). An α7 nicotinic and GABAB receptor-mediated pathway controls acetylcholine release in the tripartite neuromuscular junction. J. Physiol..

[19] Angaut-Petit D., Molgo J., Connold A.L., Faille L. (1987). The levator auris longus muscle of the mouse: A convenient preparation for studies of short- and long-term presynaptic effects of drugs or toxins. Neurosci. Lett..

[20] Benedetti B., Matyash V., Kettenmann H. (2011). Astrocytes control GABAergic inhibition of neurons in the mouse barrel cortex. J. Physiol..

[21] Liu Y.B., Guo J.Z., Chiappinelli V.A. (2007). Nicotinic receptor-mediated biphasic effect on neuronal excitability in chick lateral spiriform neurons. Neuroscience.

[22] Fucile S., Sucapane A., Eusebi F. (2005). Ca^2+^ permeability of nicotinic acetylcholine receptors from rat dorsal root ganglion neurones. J. Physiol..

[23] Liou J.C., Kang K.H., Chang L.S., Ho S.Y. (2006). Mechanism of beta-bungarotoxin in facilitating spontaneous transmitter release at neuromuscular synapse. Neuropharmacology.

[24] Kato M., Tanaka N., Usui S., Sakuma Y. (2006). The SK channel blocker apamin inhibits slow afterhyperpolarization currents in rat gonadotropin-releasing hormone neurones. J. Physiol..

[25] Dibattista M., Mazzatenta A., Grassi F., Tirindelli R., Menini A. (2008). Hyperpolarization-activated cyclic nucleotide-gated channels in mouse vomeronasal sensory neurons. J. Neurophysiol..

[26] Massote P.D., Pinheiro A.C., Fonseca C.G., Prado M.A., Guimarães A.L., Massensini A.R., Gomez M.V. (2008). Protective effect of retinal ischemia by blockers of voltage-dependent calcium channels and intracellular calcium stores. Cell Mol. Neurobiol..

[27] Bazzy A.R. (1994). Developmental changes in rat diaphragm endplate response to repetitive stimulation. Brain research. Dev. Brain Res..

[28] Bogacheva P.O., Potapova D.A., Gaydukov A.E. (2025). Sortilin and L-type Calcium Channels May be Involved in the Unusual Mechanism of proBDNF Signaling in Regenerating Mouse Neuromuscular Junctions. Neurochem. Res..

[29] McLachlan E.M., Martin A.R. (1981). Non-linear summation of end-plate potentials in the frog and mouse. J. Physiol..

[30] Betz W.J., Bewick G.S. (1992). Optical analysis of synaptic vesicle recycling at the frog neuromuscular junction. Science.

[31] Heredia D.J., Schubert D., Maligireddy S., Hennig G.W., Gould T.W. (2016). A Novel Striated Muscle-Specific Myosin-Blocking Drug for the Study of Neuromuscular Physiology. Front. Cell. Neurosci..

[32] Gafurova C.R., Tsentsevitsky A.N., Fedorov N.S., Khaziev A.N., Malomouzh A.I., Petrov A.M. (2024). β2-Adrenergic Regulation of the Neuromuscular Transmission and Its Lipid-Dependent Switch. Mol. Neurobiol..

[33] Sanes J.R., Lichtman J.W. (1999). Development of the vertebrate neuromuscular junction. Annu. Rev. Neurosci..

[34] Silveira P.E., Lima R.F., Guimarães J.D., Molgó J., Naves L.A., Kushmerick C. (2015). Ryanodine and inositol triphosphate receptors modulate facilitation and tetanic depression at the frog neuromuscular junction. Muscle Nerve.

[35] Estep C.M., Galtieri D.J., Zampese E., Goldberg J.A., Brichta L., Greengard P., Surmeier D.J. (2016). Transient Activation of GABAB Receptors Suppresses SK Channel Currents in Substantia Nigra Pars Compacta Dopaminergic Neurons. PLoS ONE.

[36] Stocker M. (2004). Ca(2+)-activated K+ channels: Molecular determinants and function of the SK family. Nature reviews. Neuroscience.

[37] Oldham W.M., Hamm H.E. (2008). Heterotrimeric G protein activation by G-protein-coupled receptors. Nature reviews. Mol. Cell Biol..

[38] Zamponi G.W., Currie K.P. (2013). Regulation of Ca(V)2 calcium channels by G protein coupled receptors. Biochim. Et Biophys. Acta.

[39] Bogaj K., Urban-Ciecko J. (2025). Inhibition of BK channels by GABAb receptors enhances intrinsic excitability of layer 2/3 vasoactive intestinal polypeptide-expressing interneurons in mouse neocortex. J. Physiol..

[40] Ramakrishna Y., Sadeghi S.G. (2020). Activation of GABAB receptors results in excitatory modulation of calyx terminals in rat semicircular canal cristae. J. Neurophysiol..

[41] Rosato Siri M.D., Uchitel O.D. (1999). Calcium channels coupled to neurotransmitter release at neonatal rat neuromuscular junctions. J. Physiol..

[42] Nadal L., Garcia N., Hurtado E., Simó A., Tomàs M., Lanuza M.A., Cilleros V., Tomàs J.M. (2016). Synergistic Action of Presynaptic Muscarinic Acetylcholine Receptors and Adenosine Receptors in Developmental Axonal Competition at the Neuromuscular Junction. Dev. Neurosci..

[43] Darabid H., St-Pierre-See A., Robitaille R. (2018). Purinergic-Dependent Glial Regulation of Synaptic Plasticity of Competing Terminals and Synapse Elimination at the Neuromuscular Junction. Cell Rep..

[44] Thompson W.J. (1985). Activity and synapse elimination at the neuromuscular junction. Cell. Mol. Neurobiol..

[45] Colman H., Nabekura J., Lichtman J.W. (1997). Alterations in synaptic strength preceding axon withdrawal. Science.

[46] Tomàs J., Garcia N., Lanuza M.A., Santafé M.M., Tomàs M., Nadal L., Hurtado E., Simó A., Cilleros V. (2017). Presynaptic Membrane Receptors Modulate ACh Release, Axonal Competition and Synapse Elimination during Neuromuscular Junction Development. Front. Mol. Neurosci..

[47] Wang M., Kleele T., Xiao Y., Plucinska G., Avramopoulos P., Engelhardt S., Schwab M.H., Kneussel M., Czopka T., Sherman D.L. (2021). Completion of neuronal remodeling prompts myelination along developing motor axon branches. J. Cell Biol..

[48] Corell M., Wicher G., Radomska K.J., Dağlıkoca E.D., Godskesen R.E., Fredriksson R., Benedikz E., Magnaghi V., Fex Svenningsen A. (2015). GABA and its B-receptor are present at the node of Ranvier in a small population of sensory fibers, implicating a role in myelination. J. Neurosci. Res..

[49] Magnaghi V., Ballabio M., Camozzi F., Colleoni M., Consoli A., Gassmann M., Lauria G., Motta M., Procacci P., Trovato A.E. (2008). Altered peripheral myelination in mice lacking GABAB receptors. Mol. Cell. Neurosci..

[50] Malomuzh A.I., Nurullin L.F., Nikolsky E.E. (2015). Immunohistochemical Evidence of the Presence of Metabotropic Receptors for γ-Aminobutyric Acid at the Rat Neuromuscular Junctions. Dokl. Biochem. Biophys..

[51] Nurullin L.F., Nikolsky E.E., Malomouzh A.I. (2018). Elements of Molecular Machinery of GABAergic Signaling in the Vertebrate Cholinergic Neuromuscular Junction. Acta Histochem..

[52] Kilb W., Kirischuk S. (2022). GABA Release from Astrocytes in Health and Disease. Int. J. Mol. Sci..

[53] Nayak T.K., Chakraborty S., Zheng W., Auerbach A. (2016). Structural correlates of affinity in fetal versus adult endplate nicotinic receptors. Nat. Commun..

[54] Li H., Teng J., Hibbs R.E. (2024). Structural switch in acetylcholine receptors in developing muscle. Nature.

[55] Brown M.C., Jansen J.K., Van Essen D. (1976). Polyneuronal innervation of skeletal muscle in new-born rats and its elimination during maturation. J. Physiol..

[56] Balice-Gordon R.J., Lichtman J.W. (1990). In vivo visualization of the growth of pre- and postsynaptic elements of neuromuscular junctions in the mouse. J. Neurosci..

[57] Marques M.J., Conchello J.A., Lichtman J.W. (2000). From plaque to pretzel: Fold formation and acetylcholine receptor loss at the developing neuromuscular junction. J. Neurosci..

